# Supplementary material for: A Novel Three-Polysaccharide Blend In Situ Gelling Powder for Wound Healing Applications
Source: Pharmaceutics. 2021 Oct 14;13(10):1680. doi: 10.3390/pharmaceutics13101680 (PMC8541204; doi:10.3390/pharmaceutics13101680)
Supplement: Supplementary file 1 [file pharmaceutics-13-01680-s001.zip › pharmaceutics-1385186-supplementary.pdf]

pharmaceutics

MDPI

Supplementary Materials: A Novel Three-Polysaccharide Blend In Situ Gelling Powder for Wound Healing Applications

Chiara Amante, Tiziana Esposito, Pasquale Del Gaudio, Veronica Di Sarno, Amalia Porta, Alessandra Tosco, Paola Russo, Luigi Nicolais and Rita P. Aquino

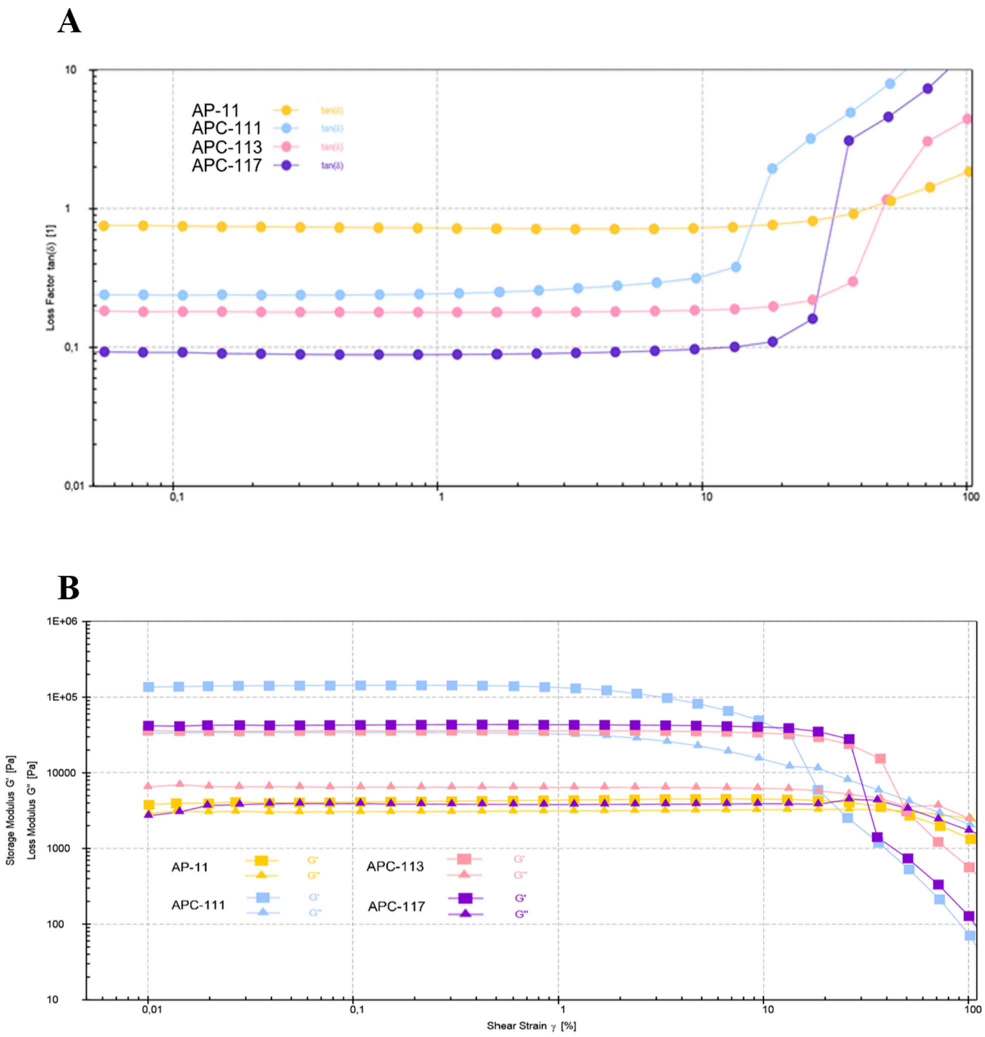

Figure S1. Rheological studies on alginate-pectin and alginate-pectin-chitosan blend *in situ* formed hydrogel after contact with simulated wound fluid; Panel A: tan δ, Panel B: elastic and plastic modulus.

Table S1. Effect polymeric blend and doxycycline loading on particle size distribution.

**Sample code**	**Alginate/Pectin/Chitosan Ratio**	**Doxycycline (w/w)**	**d_{10} (µm)**	**d_{50} (µm)**	**d_{90} (µm)**	**span**
AP-11	1:1		0.87	2.02	4.06	1.58
AP-31	3:1	-	0.93	2.09	4.76	1.83
AP-13	1:3		1.23	2.73	5.36	1.51
AP-11-1D	1:1	1.0%	1.11	2.08	4.17	1.47
AP-11-2D	1:1		1.02	2.15	4.36	1.55
AP-31-2D	3:1	2.0%	1.08	2.19	4.65	1.63
AP-13-2D	1:3		0.89	2.65	5.02	1.56
APC-111	1:1:1		1.15	3.12	6.53	1.72
APC-113	1:1:3	-	1.19	2.75	5.02	1.39
APC-117	1:1:7		1.13	2.47	4.74	1.46
APC-111-1D	1:1:1		1.02	3.21	5.73	1.47
APC-113-1D	1:1:3	1.0%	0.98	2.63	4.87	1.48
APC-117-1D	1:1:7		0.75	2.18	4.31	1.63
APC-111-2D	1:1:1		1.17	3.42	5.86	1.37
APC-113-2D	1:1:3	2.0%	1.08	2.58	4.52	1.33
APC-117-2D	1:1:7		0.97	2.37	4.51	1.49
